# Sub region-specific modulation of synchronous neuronal burst firing after a kainic acid insult in organotypic hippocampal cultures

**DOI:** 10.1186/1471-2202-9-59

**Published:** 2008-07-02

**Authors:** Christopher A Reid, Brendan EL Adams, Damian Myers, Terence J O'Brien, David A Williams

**Affiliations:** 1 Department of Physiology, The University of Melbourne, Melbourne, Australia; 2 Ion Channels and Disease Group, Howard Florey Institute, Melbourne, Australia; 3 Department of Medicine, Surgery and Neurology, The University of Melbourne, Melbourne, Australia

## Abstract

**Background:**

Excitotoxicity occurs in a number of pathogenic states including stroke and epilepsy. The adaptations of neuronal circuits in response to such insults may be expected to play an underlying role in pathogenesis. Synchronous neuronal firing can be induced in isolated hippocampal slices and involves all regions of this structure, thereby providing a measure of circuit activity. The effect of an excitotoxic insult (kainic acid, KA) on Mg^2+^-free-induced synchronized neuronal firing was tested in organotypic hippocampal culture by measuring extracellular field activity in CA1 and CA3.

**Results:**

Within 24 hrs of the insult regional specific changes in neuronal firing patterns were evident as: (i) a dramatic *reduction *in the ability of CA3 to generate firing; and (ii) a contrasting *increase *in the frequency and duration of synchronized neuronal firing events in CA1. Two distinct processes underlie the increased propensity of CA1 to generate synchronized burst firing; a lack of ability of the CA3 region to 'pace' CA1 resulting in an increased frequency of synchronized events; and a change in the 'intrinsic' properties limited to the CA1 region, which is responsible for increased event duration. Neuronal quantification using NeuN immunoflurescent staining and stereological confocal microscopy revealed no significant cell loss in hippocampal sub regions, suggesting that changes in the properties of neurons within this region were responsible for the KA-mediated excitability changes.

**Conclusion:**

These results provide novel insight into adaptation of hippocampal circuits following excitotoxic injury. KA-mediated disruption of the interplay between CA3 and CA1 clearly increases the propensity to synchronized firing in CA1.

## Background

Excitotoxicity is linked to many disease states, the consequences of which are thought to be critical to the pathogenic process [1-8]. Kainic acid (KA), a glutamate receptor agonist is frequently used to model such insults both *in vivo *and *in vitro *[9-16]. Excitotoxicity induced by KA initiates a cascade of events at multiple levels, including neuronal death, transcriptional changes in ion channels and altered synaptic plasticity [3,4,10,12,15,17-19]. The ultimate pathogenic outcome of an excitotoxic insult is likely to be reflected in changes in neuronal network activity. Identifying changes in network properties is therefore important to our overall understanding of underlying pathogenesis caused by excitotoxcity.

The hippocampal slice preparation is widely used to investigate the cellular and synaptic mechanisms that underlie synchronized network events. In isolated hippocampal slices, network firing is initiated in the CA3 region and propagates along the output pathway of the Schaffer collaterals to the CA1 region where it synchronizes neuronal firing, although CA1-led bursts have been reported [20-26]. Cells within each sub region of the hippocampus are highly heterogeneous and respond quite distinctly to a KA insult. Changes in the hyperpolarization-activated, cyclic nucleotide-gated channels (HCN) that mediate a mixed cationic conductance (I_h_) showcase this well. Heterogeneous changes in subtype and extent of HCN mRNA are evident in *in vivo *[3] and *in vitro *[27] KA models. These changes occur within 24 hours and are likely to impact on network activity in a regional specific manner. KA-mediated changes are unlikely to be limited to HCN, with transcriptional changes in other proteins and structural changes (eg. synaptic reorganization) also able to impact on measures of hippocampal excitability [5,28-32]. Furthermore, alteration in network properties in one subregion are known to modulate network activity in connecting regions [33]. Synchronized network firing is an encompassing parameter that integrates these various plastic changes.

In this paper, we investigate the impact of KA-mediated excitotoxicity on synchronized network firing in an *in vitro *model. Within 24 hours following a KA insult the hippocampal slice cultures were essentially unable to generate synchronized neuronal firing in CA3 but recovered this ability by 7 days. Simultaneously, slice cultures showed an increase in synchronized burst firing in CA1 consistent with a hyperexcitable phenotype. This excitability persisted over time. Two distinct mechanisms drive different aspects of the increased propensity to synchronized burst firing in CA1, namely a loss of the 'extrinsic' modulation by the CA3 affects burst timing, and an 'intrinsic' change limited to the CA1 sub region affects burst duration.

## Results

### Organotypic hippocampal slice cultures exhibit synchronous bursts led by CA3

**Figure 1:**
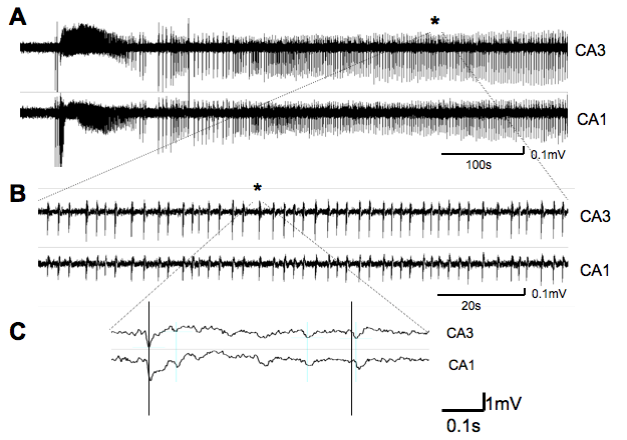
**Mg^2+^-free induced synchronized neuronal activity is initiated in CA3 of organotypic hippocampal cultures.** (A) Extracellular recordings from CA1 and CA3 regions. After a short delay Mg^2+^-free ACSF induces a burst of firing in both regions that settles into prolonged rhythmic and synchronized events. (B) Expanded region of the trace from the region indicated by asterix. (C) Further time scale expansion (centered on asterix in B) reveals that CA3 firing precedes (vertical lines) activity in CA1 suggesting that under normal conditions synchronized events are paced from the CA3 region.

Synchronous discharges can be observed in Mg^2+^-free ACSF in organotypic hippocampal slice cultures [24]. The removal of Mg^2+ ^from the superfusate is thought to unblock NMDA receptors, and the effect of Mg^2+ ^removal is largely reversed by NMDA antagonists [34-37]. In the current study we monitored extracellular potentials in CA1 and CA3 regions before, and following, the addition of Mg^2+^-free ASCF to 7 day-old hippocampal cultures (Fig. 1A). During 30 min superfusion with normal ACSF (containing Mg^2+^) no spontaneous burst activity was recorded from slices (n = 119). After 10 – 20 min Mg^2+^-Free ACSF superfusion the slice cultures exhibited a sharp increase in excitability characterized by rapid burst firing (tonic-like phase) before settling into a rhythmic firing pattern (clonic-like phase) that could be sustained for extended periods (Fig. 1A). Previous studies have noted 'seizure-like events' or 'electrographic seizures' in hippocampal slice preparations progress through specific stages beginning as rapid burst firing and developing to rhythmic burst firing as described above [26,38]. In order to have consistency between recordings and slices measurements of burst parameters began 2 minutes after the onset of the rhythmic firing phase of events and continued for a minimum of 30 bursts, during which the parameters measured were relatively consistent. Firing in both the CA3 and CA1 was highly synchronized. However, firing in the CA3 region consistently preceded that of the CA1 region by an average of 15.2 ± 0.42 ms (n = 54 spikes/slice, 18 slices) suggesting CA3 region generation, or pacing, of the firing in these cultures (Fig. 1C). The mean inter-burst interval (IBI) was 3.16 ± 0.23 s with a coefficient of variance (cV_IBI_), a measure of the regularity of timing of synchronized firing, of 0.19 ± 0.02, indicating the burst timing was quite consistent during the recording period, while the mean burst duration was 0.77 ± 0.15 s. The frequency of spikes within a burst was 7.2 ± 0.8 Hz.

### Kainic acid (KA) treatment dampens CA3 burst firing but increases CA1 excitability

**Figure 2:**
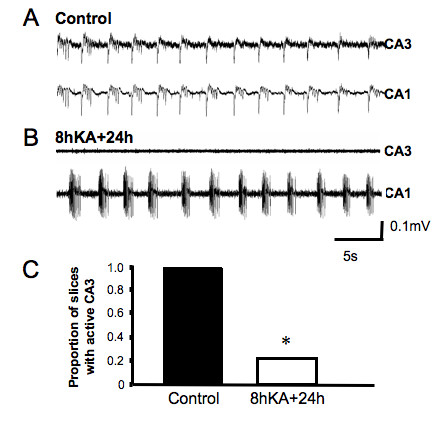
**KA insult has a regionalized impact on excitability within the hippocampal slice culture.** (A) Rhythmic synchronized neuronal events typically seen under Mg^2+^-free conditions. Activity is clearly evident in both CA1 and CA3 regions. (B) 24 hours following a KA insult, synchronized neuronal events are only recorded in the CA1 region. (C) Proportion of hippocampal slices showing CA3 firing 24 hours following KA insult, (* p < 0.05, n = 19, χ^2 ^test).

To explore the impact of excitotoxicity on the ability to develop burst firing in both CA1 and CA3 regions of the hippocampus we treated the cultures with KA (6 μM) for 8 hours. Following this insult cultures were incubated in normal medium for at least 16 hours. Extracellular recordings were made from CA1 and CA3 regions of treated cultures in Mg^2+^-free ACSF within 24 hours of termination of KA treatment (termed 24 hour time-point herein) in order to examine early post-excitotoxic changes. A dramatic reduction in the ability of the CA3 region to generate burst firing was observed 24 hours after KA treatment (Fig. 2). Indeed, in 15 of 19 slices analyzed (79%) synchronized firing was not evident in this region (p < 0.001). In contrast, burst firing persisted in the CA1 region.

### Significant cell death does not account for a loss of CA3 burst firing at the 24 hour time-point following KA treatment

**Figure 3:**
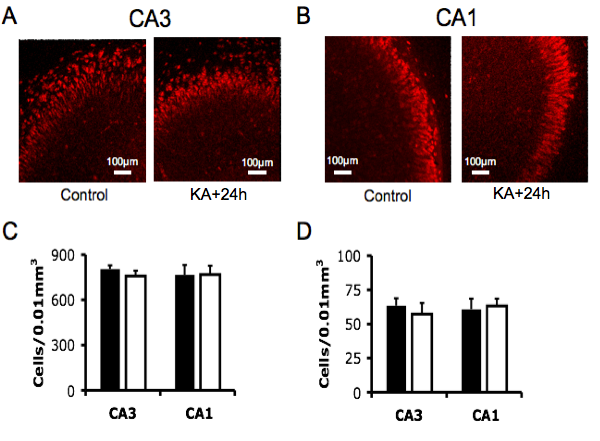
**Immunohistochemistry reveals significant cell death is not present at early time-points post KA.** (A) Micrograph of NeuN-positive staining pyramidal layer cells in CA3 and (B) CA1. (C) Cell density of NeuN-positive staining pyramidal layer cells in CA3 and CA1. Solid bars are controls, open bars are 24 hours following KA treatment (mean ± SEM, p > 0.05, n = 6, t-test). (D) Cell density of NeuN-positive staining stratum lucidium and stratum radiatum cells in CA3 and stratum lacunosum-moleculare and stratum radiatum CA1. Solid bars are controls, open bars are 24 hours following KA treatment (mean ± SEM, p > 0.05, n = 6, t-test).

Imaging of NeuN (neuron specific antibody) labeled cultures revealed that the absence of CA3 activity was not due to loss of CA3 pyramidal cells at the 24 hour time-point (Fig. 3). Differences in the labeling of control and treated cultures were indiscernible with quantification showing no significant difference in neuronal cell count. NeuN-positive cell count in the pyramidal layer was 805 ± 24 cells/0.01 mm^3 ^(control) and 758 ± 36 cells/0.01 mm^3 ^(KA) in the CA3 (p = 0.296, n = 6) and 766 ± 64 cells/0.01 mm^3 ^(control) and 767 ± 59 cells/0.01 mm^3 ^(KA) in the CA1 (p = 0.99, n = 6, Fig. 3C). Similarly, in the stratum lucidum/stratum radiatum of CA3 and stratum lacunosum-moleculare/stratum radiatum of CA1, that predominantly consist of inhibitory interneurons [39-42], NeuN-positive cell count was not affected by KA treatment: 64 ± 5 cells/0.01 mm^3 ^(control) and 57 ± 8 cells/0.01 mm^3 ^(KA) in the CA3 (p = 0.53, n = 6) and 61 ± 8 (control) and 63 ± 5 cells/0.01 mm^3 ^(KA) in the CA1 (p = 0.80, n = 6, Fig. 3D). Further, cell counts 7 days after insult also showed no significant difference between treated and control age-matched cultures. Cell death over time is expected in hippocampal slice cultures and the 7 day results reflect this. Neu-N-positive pyramidal cell count was 503 ± 60 cells/0.01 mm^3 ^(control) and 492 ± 43 cells/0.01 mm^3 ^(KA) in the CA3 (p = 0.61, n = 6), while stratum lucidium/stratum radiatum count was 49 ± 6 cells/0.01 mm^3 ^(control) and 62 ± 10 cells/0.01 mm^3 ^(KA) (p = 0.31, n = 6). Neu-N-positive pyramidal neurons in the CA1 was 578 ± 52 cells/0.01 mm^3 ^(control) and 556 ± 47 cells/0.01 mm^3 ^(KA) (p = 0.59, n = 6) and 74 ± 12 cells/0.01 mm^3 ^(control) and 64 ± 8 cells/0.01 mm^3 ^(KA) in CA1 stratum lacunosum-moleculare/stratum radiatum (p = 0.34, n = 6). This data establishes that a loss of cells cannot account for the ablation of CA3 activity, as neurons are present at both time points with no significant difference between treated and untreated cultures.

### Increased CA1 excitability follows KA treatment

**Figure 4:**
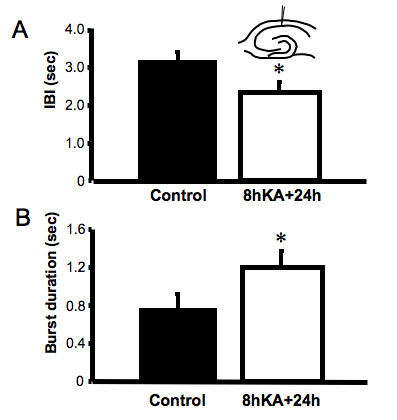
**Increases in CA1 excitability 24 hours following a KA insult.** (A) Inter-burst interval (IBI) of synchronized events in CA1 (mean ± SEM, * p < 0.05, n = 8, t-test). *Insert*, A schematic representation of the electrode placement in this experiment. (B) Duration of bursts in the CA1 region following KA insult (mean ± SEM, * p < 0.05, n = 8, t-test).

At the 24 hour time-point the CA1 region also showed a marked increase in the time spent in burst firing, due both to changes in the rate of occurrence and duration of burst events. The IBI of bursting present in CA1 region was significantly reduced after KA treatment from 3.16 ± 0.23 s to 2.32 ± 0.21 s (p < 0.03, n = 8, Fig. 4A), indicating an increased rate of occurrence of synchronized bursts following the insult. The cV_IBI _was unchanged at 0.17 ± 0.02 s, suggesting that while the pacing of bursts is altered by KA, the control of the consistency of timing of synchronized firing was maintained in the CA1 region. The duration of bursts increased after KA treatment from 0.77 ± 0.15 s to 1.19 ± 0.18 s (p < 0.05, n = 8, Fig. 4B). The frequency of spikes within a burst was not different from control (6.7 ± 0.23 Hz). These results suggest that while KA-induced changes resulted in dramatic dampening of excitability in the CA3, a paradoxical excitatory effect was evident in the CA1 region. We next asked if the increase in CA1 region excitability following the KA insult was a result of removal of the modulatory input from CA3 or a change in the intrinsic neuronal firing properties limited to the CA1 region.

### Separation of CA3 increases the rate of occurrence of burst firing in the CA1 region without changing burst duration

**Figure 5:**
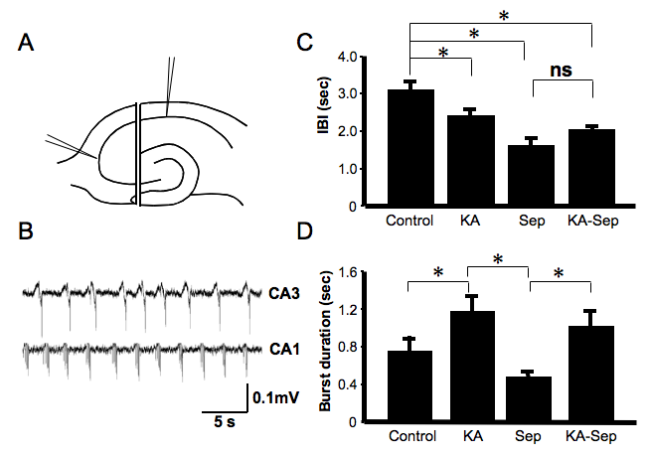
**Effects on synchronized neuronal events of acute surgical separation of CA3 from CA1 illustrates the importance of CA3 pacing on CA1 firing.** (A) Schematic diagram showing the surgical separation of CA3 from the remaining slice. (B) Synchronized neuronal firing under Mg^2+^-free conditions in slices is disrupted following CA3 separation. Events in CA1 and CA3 are no longer in phase or of similar frequency. (C) IBI of synchronized events in CA1 following separation from CA3 in both control and KA-treated slices (mean ± SEM, * p < 0.05, n = 6, one-way ANOVA). (D) Duration of bursts in CA1 following CA3 removal in both control and KA-treated slices (mean ± SEM, * p < 0.05, n = 6, one-way ANOVA).

Our results, and those of several other studies, indicated that under normal conditions burst firing in isolated hippocampal slice is paced by the CA3 region. To test the impact of this pacing on CA1 excitability in slice cultures we surgically separated the CA3 region from the remaining hippocampal culture (Fig. 5A). Previous studies in acute slice preparations have shown isolation of CA1 causes loss of burst activity [26,37]. Here bursting in the CA1 region persisted, however lost synchronicity with CA3 region activity. Furthermore, the events were more frequent (Fig. 5B), with a significantly reduced IBI (1.64 ± 0.19 s, n = 6) relative to both control (3.16 ± 0.23 s, p < 0.005) and to intact KA-treated slices (2.32 ± 0.21 s, p < 0.03, Fig. 5C). This increased rate of occurrence of bursts in the CA1 region following separation is consistent with, but greater in magnitude to, that observed following KA treatment. The cV_IBI _was increased following deafferentation (0.34 ± 0.03) compared to both control (0.19 ± 0.02) and KA-treated slices (0.17 ± 0.02). This suggests that under standard conditions burst firing can occur independently in the CA1 region, but the regularity of this activity is determined by the CA3 input. Interestingly, in contrast to the increase seen following KA treatment, burst duration tended to decrease following deafferentation (0.49 ± 0.06 s vs. 0.76 ± 0.15 s, n = 6, p = 0.06, Fig. 5D). This suggests that dampening of the CA3 region firing following KA treatment cannot explain this component of the increased CA1 region excitability.

### Increases in burst duration in the CA1 region following KA insult are maintained in segregated slices

The impact of CA3 segregation on CA1 region excitability was also investigated in slices treated with KA. The IBI of the separated CA1 region did not change (2.05 ± 0.49 s, n = 6) from that measured in intact KA-treated slices (2.32 ± 0.21 s, n = 6. p = 0.63, Fig. 5C). Comparison of this with the reduction in IBI in the CA1 region following separation of untreated slices (1.64 ± 0.19 s, n = 6, p < 0.005), suggests that the reduced IBI of burst events following KA insult is most likely due to a reduction in pacing from the CA3 region.

In untreated cultures, CA1 burst duration tended to decrease when the CA3 was separated from the slice while in KA-treated cultures the duration of the bursts increased. Intriguingly, in KA-treated slices where the CA3 was segregated, the increase in CA1 burst duration was maintained (1.03 ± 0.17 s vs. 1.19 ± 0.17 s, n = 6, Fig. 5D). Therefore, increases in the burst duration post-KA treatment cannot be explained by removal of CA3 input. These results suggest that KA treatment alters the properties of neuronal firing within either the CA1 and/or DG regions of the hippocampal slice to increase burst duration.

### DG segregation does not alter CA1 firing

The ability of the DG to display synchronized firing in Mg^2+^-free ACSF has been reported [24]. The significant reciprocal projections present in hippocampal slice cultures between CA1 and DG raise the possibility that changes in the intrinsic properties of the DG may modulate changes in CA1 burst duration [11]. To establish the relationship between CA1 and DG we recorded from both regions in CA3-deafferented slices. CA1 firing preceded the DG by an average of 15.25 ± 2.34 ms (n = 6), indicating that in the absence of the CA3 region, CA1 paces synchronized firing. However, it remains possible that a reciprocal interplay between CA1 and DG could influence burst duration in CA1 region.

**Figure 6:**
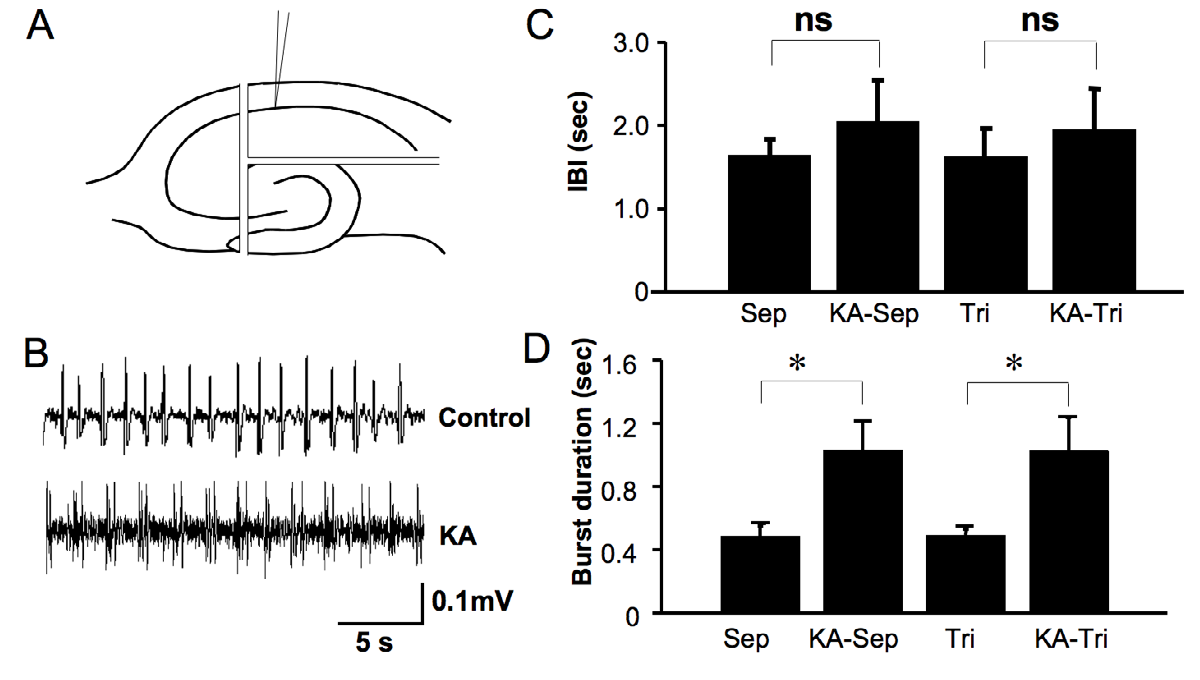
**Isolated CA1 region slices behave identically to CA1/DG slices, ruling out an influence of DG on CA1 region excitability following a KA insult.** (A) Cartoon showing the surgical separation of CA1. (B) Synchronized neuronal firing under Mg^2+^-free conditions in a slice following separation. (C) IBI of synchronized events in CA1 following separation and trisectioning (mean ± SEM, n = 6, one-way ANOVA). (D) Burst duration in CA1 following separation (mean ± SEM, * = p < 0.05, ns = p > 0.05, n = 6, one-way ANOVA. Sep = CA3 separation/deafferentation, Tri = trisectioned to leave only CA1).

To test this we isolated CA1 from all other hippocampal regions immediately prior to recording Mg^2+^-free induced synchronized firing. The IBI (1.63 ± 0.33 s vs. 1.64 ± 0.19 s, n = 6) and burst duration (0.49 ± 0.03 s vs. 0.49 ± 0.06 s, n = 6) of isolated CA1 regions developed activity that was no different to that of slices that still contained the DG. Therefore, it is unlikely that DG influences the observed changes in CA1 excitability. This was confirmed by evaluation of the impact of KA treatment on isolated CA1 region firing (Fig. 6). Isolation of the CA1 region did not change the IBI (Fig. 6C). However, the increase in burst duration was maintained (1.03 ± 0.21 s vs. 0.49 ± 0.03, p = 0.01, n = 6, Fig. 6D). It is also similar in duration to CA1 activity recorded from CA3 deafferentation slices (1.03 ± 0.18, n = 6) and whole slices (1.19 ± 0.18, n = 8) following KA treatment. Therefore, increased CA1 excitability in this preparation is due to both a reduction in the 'extrinsic' pacing input from CA3 (resulting in more frequent bursts) and changes to 'intrinsic' properties limited to the CA1 sub-region (resulting in increased duration of bursts) following a KA insult.

### Increases in CA1 region burst duration, but not rate of occurrence, are sustained for up to 7 days following KA insult

**Figure 7:**
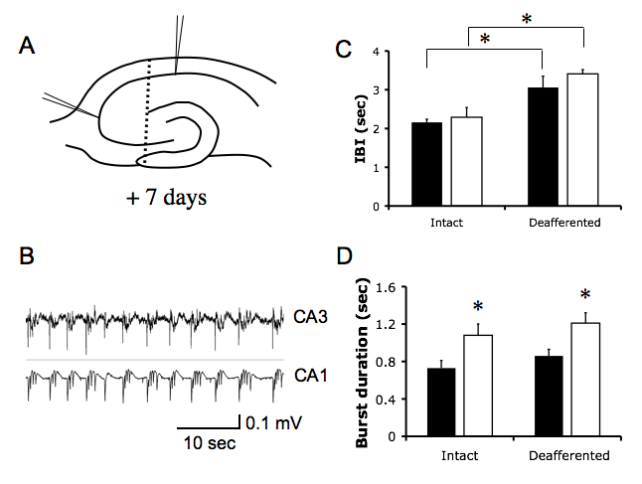
**CA1 hyperexcitability is maintained 7 days after KA insult.** (A) Schematic showing electrode placement. Dotted line indicates surgical separation of CA3. (B) Example of synchronized neuronal burst firing under Mg^2+^-free conditions at the 7 day post KA time-point (C) IBI in cultures 7 days after KA insult and age-matched controls prior to and after surgical separation of CA3 (* p < 0.05, n = 6, ANOVA). (D) Burst duration in CA1 7 days after KA treatment, prior to and after CA3 deafferentation (* p < 0.05, n = 6, ANOVA). Closed bars are controls, open bars are 8 h KA-treated +7d.

To examine whether the functional changes were chronically sustained following acute KA-treatment, we recorded extracellular field potentials in CA1 and CA3 regions 7 days after KA insult. The ability of CA3 region to generate firing under Mg^2+^-free conditions had returned at this later time-point with burst amplitudes consistent with control cultures (92%, n = 12, Fig. 7B). The IBI at this time was not significantly different from that recorded in control, non-treated cultures of identical age (2.29 ± 0.25 s, n = 8 vs. 2.16 ± 0.08 s, n = 6, Fig. 7C). This result is consistent with the premise that a return of CA3 pacing function accounts for the normalization of the rate of occurrence of burst-firing events in CA1. In contrast, the KA-induced increases in burst duration within the CA1 were maintained at this later time-point (1.08 ± 0.12 s, n = 6 vs. 0.73 ± 0.08 s, n = 6, P < 0.05, Fig. 7D). The duration of bursts was not different for CA3 and CA1 regions in KA-treated slices.

To confirm the importance on CA3 input in pacing burst frequency we again separated the CA3 from the CA1 just prior to recording at this later time-point. Following deafferentation we found IBI increased in both control to (2.16 ± 0.08 s to 3.06 ± 0.29 s, p = 0.04, n = 8, Fig. 7C) and KA-treated slices (2.29 ± 0.25 s to 3.41 ± 0.11 s, p = 0.03, n = 8, Fig. 7C). Thus removal of CA3 input again resulted in a disruption in the timing of IBI. However, rather than the decrease seen early after KA insult, we now measured a significant increase in IBI over the intact slices. There remained no significant difference in IBI between treated and control slices (p = 0.63, n = 8, Fig. 7D). Conversely, removal of CA3 input had no significant impact on the burst duration in CA1 of deafferented slices compared to their intact counterparts in control (0.86 ± 0.07 s vs 0.73 ± 0.08, p = 0.08, n = 8, Fig. 7D) or treated slices (1.21 ± 0.11 s vs 1.08 ± 0.12 s, p = 0.11 n = 8, Fig. 7D). The elevated burst duration following KA treatment was maintained regardless of CA3 deafferentation (0.86 ± 0.07 vs 1.21 ± 0.11 s, p = 0.03, n = 8, Fig. 7D).

## Discussion

Here we describe three primary findings. Firstly, that a loss of CA3 pacing 'control' of CA1 can increase excitability in this region. Although not an entirely new concept [43], this is the first demonstration of such interplay between CA3 and CA1 under pathological conditions. Secondly, that a chemical (KA) rather than a surgical insult is capable of 'isolating' the CA1 region from CA3. Given that KA models excitotoxcity this result provide evidence of a novel hypothesis regarding the mechanisms underlying hippocampal epileptogenesis following an excitotoxic insult that needs further testing in other brain model systems. Finally, that the different aspects of burst behaviour (frequency of events and duration of events) can be modulated by distinct network factors.

### Increases in excitability of the CA1 region and hyperexcitable phenotypes

The properties of the CA3 region, including a propensity to bursting and abundant recurrent collaterals, make it an obvious choice for driving a hyperexcitable hippocampus [43-45]. However, hyperexcitability limited to the CA1 region is recognized [16,32,46]. An increase in CA1 pyramidal neuron axon sprouting occurs in KA- and pilocarpine-treated animals [5,28,29]. *De novo *recurrent excitatory connections established within the CA1 following an excitotoxic insult are postulated to contribute to a hyperexcitable hippocampus [11]. In this sense the organotypic culture models a hippocampus that has already formed these recurrent CA1 connections. The KA insult therefore may model a further insult under such hyperexcitable conditions, as may be expected in epilepsy cases of multiple and progressively worsening seizures. The sustained changes in burst duration we observe in this model could therefore contribute to overall hippocampal hyperexcitability. Determining the molecular and/or structural basis of a sustained increase in burst duration is therefore important.

Structurally, KA-treated organotypic slices have a greater extent of CA1 pyramidal neuron axon sprouting, a feature that could change the dynamics of synchronization [11]. However, increases in axon sprouting occur on relatively slow time scales, and as such, are unlikely to be responsible for changes noted at the 24 hour time-point. While we observe no cell death in our cultures, selective CA1 interneurons are particularly sensitive to the toxic effects of KA, potentially altering the excitation/inhibition balance to render the CA1 hyperexcitable [16,47,48]. CA1 pyramidal neurons make recurrent excitatory contacts with interneurons producing feedback inhibition, and a specific loss of this function, through either loss of interneurons or changes in the intrinsic properties of interneurons may prolong burst duration [48,49]. Burst appear to be terminated by activity-dependent depression at recurrent excitatory synapses, and IBI is predicted to be primarily due to the period of recovery from synaptic depression [50-52]. However, an alternative proposition for the pacing of IBIs is the slow build-up of excitation governed by I_h _[20,53,54]. As already noted, transcriptional changes in ion channel expression do occur on short time frames and could contribute to changes seen. Two good candidates are alterations in I_h _and T-type Ca^2+ ^channels. Increased CA1 neuronal bursting due to the *de novo *expression of Ni^2+^-sensitive T-type Ca^2+ ^channels is associated with epilepsy in the pilocarpine model of temporal lobe epilepsy (TLE) [55,56]. Similarly, increased HCN channel function in CA1 pyramidal neurons of the febrile convulsive model of TLE is predicted to enhance synchronized neuronal firing [4].

### What causes a transient decrease in CA3 excitability?

The basis of the transient absence of Mg^2+^-free synchronized firing in the CA3 region remains to be determined. KA applied for longer periods to organotypic hippocampal slices do illicit a selective degeneration of CA3 pyramidal cells [10,57], raising the possibility that rapid cell death may be responsible. However, KA insult had no effect on the count of pyramidal neurons, or stratum lucidium and stratum radiatum inhibitory interneurons at either the 24 hour or 7 day time-points in our study. This is consistent with previous studies that suggest that long-term, (12 to 48 hours) rather than short-term, (4 to 6 hours) KA exposure results in cell death in organotypic cultures at similar concentrations [7,9,10,12]. Furthermore, a return of CA3 bursting activity occurs at 7 days suggesting that neurons required to develop synchronized firing are present at the 24 hour time-point, but are unable to do so. Therefore, the acute reduction in burst activity is more likely to reflect a functional change in CA3 sub-region neurons rather than be a consequence of cell death (or cells in the process of dying). Previous studies of acute KA application have identified a wide range of hippocampal alterations, including changes in GABA_A _and GABA_B _receptor expression, matrix metalloproteinase activity, mossy fiber innervation, and expression of neuronal and glial glutamate transporters and ongoing neuronal degeneration [9,12,18,58-60] or transcriptional changes as previously discussed [3,4,56,61-63]. Interneurons play an essential role in neuronal network synchronization and disruption of their input is expected to alter CA3 burst activity [48]. Loss of GABA_B _receptor-mediated heterosynaptic depression in mossy fibres innervating the CA3, postulated to contribute to maintenance of seizure activity, has also been reported 24 hours after pilocarpine exposure [64]. Therefore changes to the intrinsic properties of excitatory or inhibitory neurons may contribute to the loss of activity in CA3.

### Potential pathophysiological implications

In rats *in vivo*, KA-induced *status epilepticus *and its sequelae are widely used to model the development of TLE [65]. *In vitro*, application of KA to organotypic slices results in many of the hallmarks of TLE, including mossy fiber sprouting, eventual hippocampal neuronal death (particularly in the CA3, dentate hilus and CA1 regions), and an enhanced propensity to synchronized neuronal firing [9,10,57]. Our results suggest that in the hippocampus, KA-mediated excitotoxic stimuli cause changes in neuronal network firing patterns that are highly region-specific. Paradoxically, reduced excitability in the CA3 region can enhance excitability in the CA1 region. Conceptually, this provides a novel means by which hippocampal excitability can be increased. In this scheme an adaptive process, presumably attempting to limit hyperexcitability in the CA3, engages or affects a mechanism resulting in an increased CA1 excitability. A similar mechanism has been proposed to unmask recurrent entorhinal seizures in intact hippocampal-entorhinal slices in which the Shaffer collateral fibers had been cut [33,43]. A potential implication of this finding relates to the progressive neuronal death in selected hippocampal subfields including CA3 noted in patients and most models of TLE. This takes on extra significance given that an increase in CA1 pyramidal neuron axon sprouting occurs in KA- and pilocarpine-treated animals [5,28,29]. A combination of these factors and 'intrinsic' changes responsible for burst duration in CA1 may contribute to a progressively worsening of seizures noted in TLE.

## Conclusion

Excitotoxicity is one of the major pathological processes underlying both acute and chronic neurological diseases, including stroke and epilepsy. Here we describe the impact on regional neuronal firing patterns in CA1 and CA3 of an acute KA-induced excitotoxic insult to hippocampal organotypic cultures. A KA insult resulted in a significant dampening of the capacity of CA3 regions to generate Mg^2+^-free induced burst neuronal firing at the 24 hours time-point. In contrast, this insult lead to increased propensity of the CA1 region to synchronized neuronal burst firing by increasing both the rate of occurrence and duration of the events. A loss of 'pacing' by CA3 of the synchronisation of CA1 accounted for the increased rate of occurrence in bursting events. Changes in the properties limited to the CA1 region were responsible for increased event duration. These findings provide a framework on which to consider potential contributors to hippocampal hyperexcitability in TLE.

## Methods

### Hippocampal slices

All procedures undertaken in this project were ratified by the University of Melbourne animal ethics committee. Transverse 300 μm slices of hippocampus were cut from 7 to 9-day-old Sprague Dawley rat pups and cultured according to published methods [66] on Millicell CM membranes (Millipore MA, USA) for 7–14 days prior to use, as described previously [67]. A subset of cultures was treated with 6 μM KA (Sigma MI, USA) for 8 hours and returned to normal medium for incubation prior to examination the following day, within 24 hours post insult. Several slices are analyzed during one day therefore the time after insult varies from approximately 16 hours to 24 hours, herein referred to this as the 24 hour time point. Physical separation of CA3/dentate gyrus (DG) regions was achieved by using a scalpel blade under a binocular dissecting microscope. All surgical interventions were made just prior to electrophysiological recordings. The selectivity of this separation procedure is highlighted [see Additional file 1].

### Extracellular field potential recordings

Slices and their supporting membranes were transferred to a recording bath where they were continually superfused with oxygenated (95%O_2_/5%CO_2_) artificial cerebrospinal fluid (ACSF) maintained at 30°–32°C with a TC-324B temperature controller (Warner Instruments, Hamden, CT). A subset of cultures recorded at room temperature yielded less robust rhythmic firing so a bath temperature of 30–32°C was used. Except where noted the ACSF contained (in mM): 120 NaCl, 3 KCl, 1 MgCl_2_, 2 CaCl_2_, 1.2 NaHPO_4_, 23 NaHCO_3_, 11 glucose. Slices were viewed through an Olympus BX51WI microscope using a XL Fluor 4X/340 NA 0.28 objective (Olympus, Japan). Simultaneous recordings from the CA3 and CA1 regions were acquired with a MiniDigi 1A two-channel acquisition system (Axon Instruments, CA, USA) using custom-made bipolar 0.025 mm polyimide insulated tungsten (Goodfellow, UK) wire electrodes, through an A-M Systems 3000 AC/DC amplifier (A-M Systems, WA, USA), Gain = 1 K, High Pass = 1 Hz, Low pass = 0.1 kHz, and recorded in AxoScope9 software (Axon Instruments, CA, USA) while sampling at 500 Hz. Recordings electrodes were alternated between region for each successive experiment ensure that differences in timing were not due to electronic delay. Mg^2+^-free ACSF perfusate, identical in composition to ACSF except for equimolar CaCl_2 _substitution for MgCl_2_, was used to promote synchronous firing, presumably by removal of NMDA receptor block, as previously described [34-36].

### Immunohistochemistry

Neurons were specifically labeled for cell counting with neuronal nuclear antigen (NeuN) antibody. Cultures were washed in phosphate-buffered saline (PBS) 0.1 M, pH 7.4 and treated with blocking buffer (BB) containing 10% foetal calf serum + 1% Triton X-100 for 30 min. Cultures were incubated overnight at 4°C with mouse anti-NeuN (Chemicon International, MAB 377, USA) at 1:1000 in BB, washed and incubated with secondary Alexa 594 donkey anti-mouse antibody (Molecular Probes) for 2 hours. Images were captured on an Olympus FV1000 confocal laser scanning microscope with spectral analysis using a UPLANSApo 20x/0.75 objective lens, running Olympus FV10 ASW Ver 1.6a image acquisition software with 3D analysis (Olympus Australia Pty Ltd). Post-hoc cell density analysis was performed using Image Pro Plus, Ver 6.2 (QImaging, Surrey, BC, Canada).

### Electrophysiological parameter measurements and statistical analysis

Inter-burst interval (IBI) was defined as the interval between the negative peaks of two adjacent events, or when bursts occurred in clusters, the interval between the first events in two consecutive clusters [see Additional file 2] [20]. Measurement of IBI were normalized between recordings and slices to start 2 minutes after bursting had established a rhythmic firing pattern and continued for a minimum of 30 bursts. Burst duration was defined as the time from the negative peaks of the first and last spikes in a burst. The frequency of spikes within a burst was determined by counting the number of spikes occurring during this time. Regularity of the occurrence of synchronized events was measured by the coefficient of variance of IBI (cV_IBI_), calculated as cV= SD/mean, determined within each recording and averaged per group. The cV_IBI _was also used to monitor the consistency of recordings to help ensure data was normalized between slices. Data is presented as mean ± standard error of the mean (SEM). A chi-squared test was performed to test proportion of slices oscillating (Fig. 2C). Differences between two means were tested by a two-tailed student's t-test (Figs. 3, 4 and 7). One-way ANOVA was used where more than two means were compared (Figs. 5 and 6). Significance level was set at p < 0.05. Slices with no evidence of burst firing were excluded from the analysis. For figures, bars represent mean ± SEM, *, P < 0.05.

## Authors' contributions

CAR initiated and designed experiments and co-wrote manuscript. BELA conducted experiments, developed methods, analyzed data and co-wrote manuscript. DM and TJOB were involved in experimental design and editing manuscript. DAW was involved in experimental design, lab direction and editing manuscript. All authors have read and approved the final article.

## Supplementary Material

Additional file 1 Supplementary Fig 1. Sectioning of cultures. (A) Photomicrograph of trisectioned hippocampal culture with isolated CA1, CA3 and DG regions. (B) Schematic showing the sectioning of cultured hippocampal slices. Click here for file

Additional file 2 Supplementary Fig 2. Measurement of IBI and burst duration. (A) Section of trace showing rhythmic burst firing in CA3 and CA1. (B) Expanded trace showing IBI measured as the interval between the negative peaks of two adjacent events (clusters) and burst duration as the time from the first to the last spikes in a burst event. Click here for file
