# Supplementary figures and images for: Sub region-specific modulation of synchronous neuronal burst firing after a kainic acid insult in organotypic hippocampal cultures
Source: BMC Neurosci. 2008 Jul 2;9:59. doi: 10.1186/1471-2202-9-59 (PMC2474631; doi:10.1186/1471-2202-9-59)

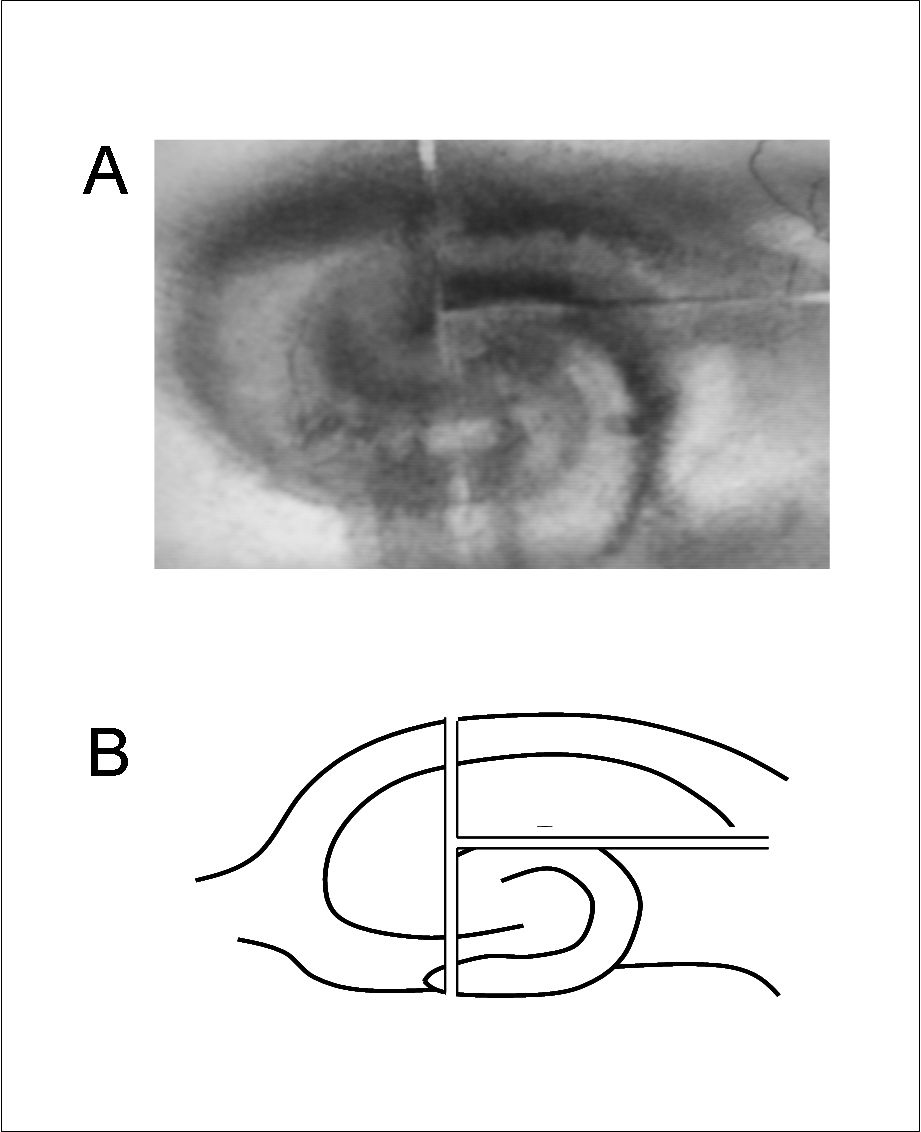

Supplement: Additional file 1 — Supplementary Fig 1. Sectioning of cultures. (A) Photomicrograph of trisectioned hippocampal culture with isolated CA1, CA3 and DG regions. (B) Schematic showing the sectioning of cultured hippocampal slices. [file 1471-2202-9-59-S1.tiff]

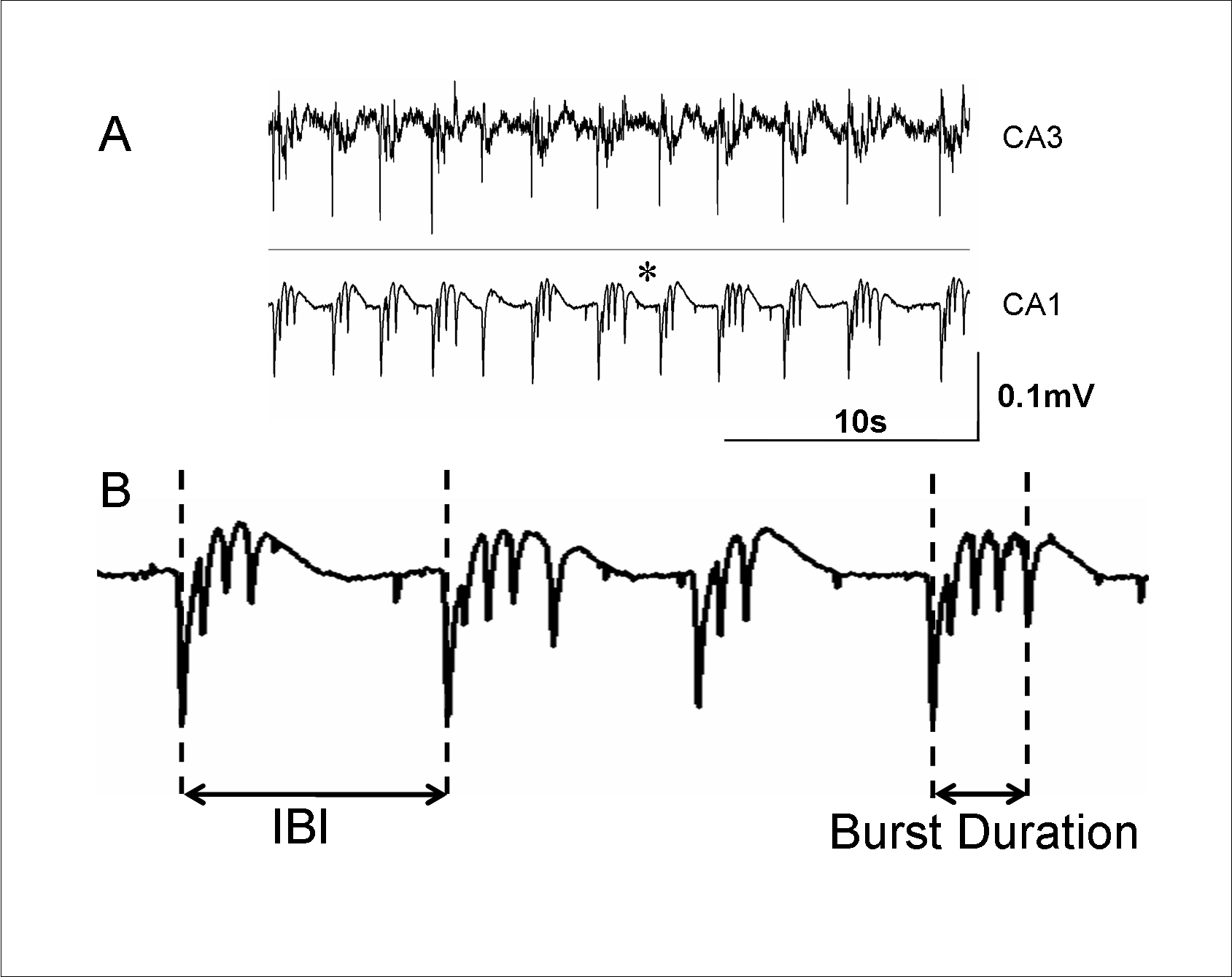

Supplement: Additional file 2 — Supplementary Fig 2. Measurement of IBI and burst duration. (A) Section of trace showing rhythmic burst firing in CA3 and CA1. (B) Expanded trace showing IBI measured as the interval between the negative peaks of two adjacent events (clusters) and burst duration as the time from the first to the last spikes in a burst event. [file 1471-2202-9-59-S2.tiff]
